# Salvianolic acid C attenuates cerebral ischemic injury through inhibiting neuroinflammation via the TLR4-TREM1-NF-κB pathway

**DOI:** 10.1186/s13020-024-00914-0

**Published:** 2024-03-11

**Authors:** Wenbo Guo, Xiaojing Xu, Yulin Xiao, Jiatian Zhang, Peiqiang Shen, Xiaoyan Lu, Xiaohui Fan

**Affiliations:** 1 Pharmaceutical Informatics Institute, College of Pharmaceutical Sciences, Zhejiang University, Hangzhou, 310058 China; 2 National Key Laboratory of Chinese Medicine Modernization, Innovation Center of Yangtze River Delta, Zhejiang University, Jiaxing, 314100 China; 3 Jinhua Institute of Zhejiang University, Jinhua, 321999 Zhejiang China; 4 Zhejiang Engineering Research Center for Advanced Manufacturing of Traditional Chinese Medicine, Huzhou, 310058 China

**Keywords:** Salvianolic acid C, Cerebral ischemic stroke, Disease network, RNA transcriptome sequencing, Neuroinflammation, TREM1

## Abstract

**Background:**

Stroke is a leading cause of mortality and disability with ischemic stroke being the most common type of stroke. Salvianolic acid C (SalC), a polyphenolic compound found in *Salviae Miltiorrhizae* Radix et Rhizoma, has demonstrated therapeutic potential in the recovery phase of ischemic stroke. However, its pharmacological effects and underlying mechanisms during the early stages of ischemic stroke remain unclear. This study aimed to examine the potential mechanism of action of SalC during the early phase of ischemic stroke using network pharmacology strategies and RNA sequencing analysis.

**Methods:**

SalC effects on infarct volume, neurological deficits, and histopathological changes were assessed in a mouse model of transient middle cerebral artery occlusion (tMCAO). By integrating RNA sequencing data with a cerebral vascular disease (CVD)-related gene database, a cerebral ischemic disease (CID) network containing dysregulated genes from the tMCAO model was constructed. Network analysis algorithms were applied to evaluate the key nodes within the CID network. In vivo and in vitro validation of crucial targets within the identified pathways was conducted.

**Results:**

SalC treatment significantly reduced infarct volume, improved neurological deficits, and reversed pathological changes in the tMCAO mouse model. The integration of RNA sequencing data revealed an 80% gene reversion rate induced by SalC within the CID network. Among the reverted genes, 53.1% exhibited reversion rates exceeding 50%, emphasizing the comprehensive rebalancing effect of SalC within the CID network. Neuroinflammatory-related pathways regulated by SalC, including the toll-like-receptor 4 (TLR4)- triggering receptor expressed on myeloid cells 1 (TREM1)-nuclear factor kappa B (NF-κB) pathway, were identified. Further in vivo and in vitro experiments confirmed that TLR4-TREM1-NF-κB pathway was down-regulated by SalC in microglia, which was essential for its anti-inflammatory effect on ischemic stroke.

**Conclusions:**

SalC attenuated cerebral ischemic injury by inhibiting neuroinflammation mediated by microglia, primarily through the TLR4-TREM1-NF-κB pathway. These findings provide valuable insights into the potential therapeutic benefits of SalC in ischemic stroke.

## Background

Stroke is one of the most serious diseases causing death and severe disability worldwide [1, 2]. In 2019, 6.55 million persons died of stroke, with 143 million disability-adjusted life years caused by the disease globally [3]. Stroke prevalence and incidence rates have increased considerably among people younger than 70 years over the last two decades, resulting in a huge burden and cost to society [3]. China has the highest estimated lifetime stroke risk (39.3%), surpassing the global average of 24.9% [4]. Ischemic stroke is the most common type, accounting for 70–80% of all stroke cases [5, 6]. Few therapeutic agents are available for the clinical treatment of ischemic stroke, except for recombinant tissue plasminogen activator (r-tPA), which was approved by the Food and Drug Administration (FDA) in 1996. However, its effectiveness is constrained by a narrow therapeutic window (3–4.5 h) and a high risk of hemorrhagic transformation. Consequently, only a small fraction (5%) of clinical patients benefits from r-tPA [7, 8]. Moreover, the early stage following stroke onset is a critical period that significantly affects patient prognosis and rehabilitation. Therefore, it is imperative to develop new therapeutic agents for the early stages of ischemic stroke.

Numerous studies have indicated that the progression of ischemic stroke is determined with multiple targets and pathways including oxidative stress, glutamate excitotoxicity, neuroinflammation, autophagy, and apoptosis [9, 10]. Neuroinflammatory responses play an essential role in cerebral ischemic injury [11]. Microglia, the resident immune cells and major mediators of the central nervous system (CNS), play critical roles in the immune regulation of neuroinflammatory diseases [12]. They are activated during the early stages of ischemic stroke and are closely associated with the inflammatory response during acute ischemic stroke [12]. Furthermore, microglial activation is the first step in the inflammatory response of the brain, followed by the infiltration of other adaptive immune cells and activation of nerve cells [13, 14]. Activated microglia move to physically surround or target dead cells, neurons, dendrites, and blood vessels to affect inflammation [15]. In animal models, an increase in infarct volume is accompanied by an increase in pro-inflammatory factors and a decrease in anti-inflammatory factors [16]. Recent studies have shown that microglial inhibition attenuates injury after cerebral infarction, and has therefore become an important intervention in the treatment of ischemic stroke [17]. Triggering receptor expressed on myeloid cells 1 (TREM1) plays an important role in the inflammatory response of microglia and is closely associated with the occurrence and development of acute ischemic stroke [18]. Studies have revealed significant up-regulation of TREM1 expression in the microglia of mice with acute ischemic stroke and subarachnoid hemorrhage. This up-regulation is associated with the severity of cerebral infarction and neurological deficits. Blocking TREM1 has been shown to enhance hippocampal cell proliferation and synaptic plasticity, which contribute to the improvement of nerve function [18, 19].

**Fig. 1:**
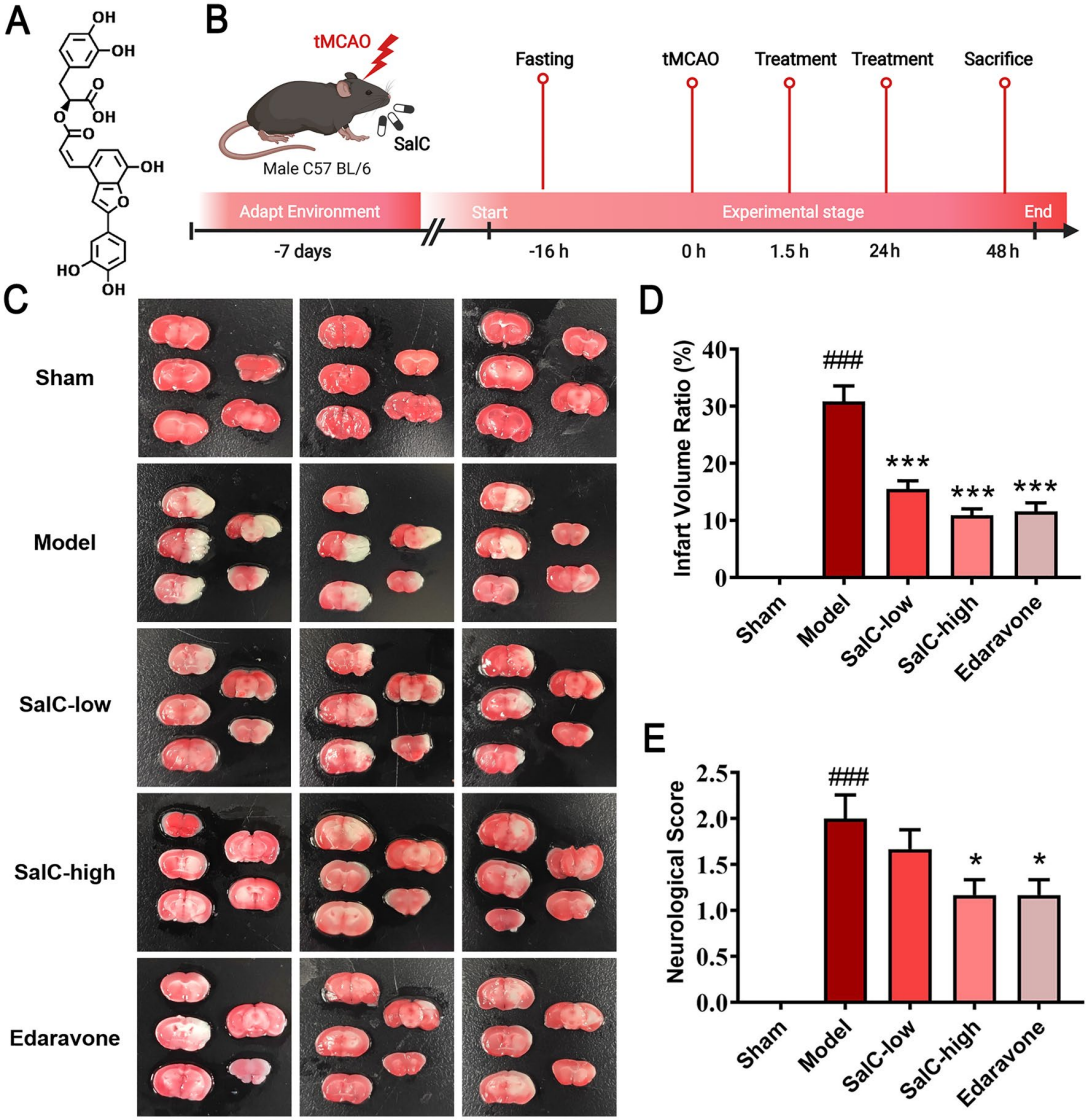
SalC reduces cerebral infarction and improves neurological function of tMCAO mice. **A** Structural formula of SalC. **B** Experimental scheme. **C** Representative TTC-stained coronary brain sections (white, infarcted area; red, non-infarcted area). **D** Statistics of the volume ratio of cerebral infarction in mice. **E** Scores of neurological deficits in different groups. Data are expressed as mean ± SEM, n = 6. The significance was determined by one-way ANOVA followed by Dunnett's posterior analysis, ^###^*P* < 0.001, *versus* the sham group; *P < 0.05, ***P < 0.001, versus the model group

Salvianolic acid C (SalC) is an organic compound mainly derived from the traditional Chinese medicine *Salviae Miltiorrhizae* Radix et Rhizoma. It consists of two units of tashinol and one unit of caffeic acid (Fig. 1A). SalC has shown to have anti-oxidative, anti-inflammatory, and anti-apoptotic activities [20, 21]. Previous reports have indicated that during the late stages of stroke, SalC can suppress inflammatory cytokine levels, promote cerebrovascular regeneration, and reduce microglial polarization [22, 23]. These findings support SalC as a potential molecular target for the treatment of late-phase stroke. However, it is essential to recognize that the acute phase following stroke is a critical period. During this early stage, inflammatory responses and neural damage play pivotal roles and have profound implications for the clinical prognosis of patients [24, 25]. Existing research has focused on the late stages of stroke; however, investigations on the acute phase of stroke can prove to be equally important. Implementing effective interventions during this early stage may significantly contribute to minimizing cerebral ischemic injury and inflammatory responses, thereby improving patient survival rates and the overall quality of life. Research on the pharmacological actions of SalC in the context of early ischemic stroke is limited, and its underlying mechanisms remain largely unexplored.

Therefore, this study aimed to bridge these gaps in the current knowledge in the field. Given the recent emergence of network pharmacology based on systems biology and polypharmacology, which uses systems pharmacology to help researchers to better understand the mechanisms of action [26–29], we used RNA-seq combined with network pharmacology approaches to investigate the therapeutic effects and mechanisms of SalC in the early stages of ischemic stroke, with a focus on microglia-mediated neuroinflammation.

## Materials and methods

### Animals

All animal experiments were approved by the Animal Care and Use Committee of the Zhejiang University School of Medicine (Zhejiang, China). Male C57BL/6J mice (8 − 10 weeks old), weighing 18–25 g, were purchased from Beijing Vital River Laboratory Animal Technology Co., Ltd. (Beijing, China). The mice were housed in a temperature-controlled animal facility (24–26 °C) under a 12 h dark/light cycle and with free access to clean water and standard diet.

### Animal model of transient middle cerebral artery occlusion (tMACO) and grouping

After seven days of acclimatization, a mouse model of cerebral ischemic stroke was induced by tMCAO based on our previously published method [30]. Briefly, mice were anesthetized with 1% sodium pentobarbital. After the mice were fixed in the supine position, an incision ~ 1 cm long was cut in the middle right of the neck to expose the right common carotid artery (CCA), internal carotid artery (ICA), and external carotid artery (ECA). Then, a silicone filament (L1800, Jialing Biotechnology Co, Ltd, Guangzhou, China) was inserted into the ICA to occlude the left middle cerebral artery (MCA). After 1.5 h, the silicone filament was gently removed to achieve postischemic reperfusion, and the neck incision was sutured. After awakening from anesthesia, the mice were assessed for neurological deficits, and mice with scores of 1–3 were selected for subsequent experiments [31]. The sham group underwent the same surgical procedure except for occlusion of the MCA. During the procedure, heat lamps (NOMOY PET, Jiaxing, China) were used to maintain the mouse rectal temperature at 37.0 ± 0.5 °C.

The mice were randomly divided into 5 groups according to body weight: (1) a sham-operated group (sham, intraperitoneally injected with normal saline at 0.1 mL/10 g body weight); (2) a model group (model, intraperitoneally injected with normal saline with the same concentration as the sham group); (3) a low-dose group of SalC (SalC-low, intraperitoneally injected with SalC at 20 mg/kg of body weight per day); (4) a high-dose group of SalC (SalC-high, intraperitoneally injected with SalC at 40 mg/kg of body weight per day); and (5) a positive control group (Edaravone, a positive drug commonly used in stroke research [32–34]), intraperitoneally injected with edaravone at 4 mg/kg of body weight per day). SalC (purity ≥ 98.0%, PubChem CID: 13991590) was obtained from Chengdu Must Bio-technology Co., Ltd. (Chengdu, China). Edaravone (batch number: 80-151203) was obtained from Nanjing Xiansheng Pharmaceutical Co., Ltd. (Nanjing, China). After reperfusion, the drugs were immediately administered to the mice. Finally, the mice were euthanized 48 h after reperfusion (Fig. 1B).

### Neurological deficit scores

Neurological deficit scores were obtained when the mice fully recovered from anesthesia and 48 h after reperfusion. Specifically, the scores were evaluated in a blinded fashion using the modified Longa’s five-point test [31], with higher scores indicating more severe neurological dysfunction (0: normal score; 4: maximal deficit score).

### 2,3,5-Triphenyltetrazolium chloride (TTC) staining

After 48 h of reperfusion, brain tissues were carefully removed and immediately frozen at − 20 °C for 20 min. Subsequently, the frozen brain was cut into five slices, each 2 mm thick, and stained with 0.25% TTC (Sigma, MO, USA) solution in the dark at 37 °C. After 30 min, the brain sections were immersed in 4% paraformaldehyde at 4 °C for 24 h. Finally, the infarct size was analyzed using Image-Pro Plus (IPP) 6.0, and the cerebral infarct volume ratio of each mouse was calculated using the following Eq. (1):

1 $$Infarct\;volume\;ratio\;(100\% )\; = \;\frac{{SUM\;of\;infarct\;area\; \times \;2\;mm^{3} }}{{SUM\;of\;total\;brain\;area\; \times \;2\;mm^{3} }}\; \times \;100\% ,$$

### Histopathological examinations and terminal deoxynucleotidyl transferase mediated dNTP nick end labeling (TUNEL) assay

After perfusion with saline, followed by 4% paraformaldehyde, brain tissues were carefully harvested and fixed with 10% formalin for 48 h. Histopathological changes were evaluated in paraffin-embedded brain sections using hematoxylin–eosin (HE) staining and detected under a microscope (E100, Nikon, Tokyo, Japan). Apoptosis in brain tissue was determined using a TUNEL assay kit (Roche, Basel, Switzerland), and TUNEL-positive cells were observed under a fluorescence microscope (ECLIPSE TI-SR; Nikon, Tokyo, Japan).

### RNA-sequencing analysis

Four mice from each group were randomly selected, and their brain tissues were used for RNA sequencing analysis. First, the total RNA was extracted by Trizol reagent after 48 h of tMCAO modeling, and the purity and integrity of RNA were examined by NanoPhotometer® spectrophotometer (IMPLEM, CA, USA). Next, an Illumina NEBNext^®^ Ultra™ RNA Library Prep Kit (NEB, USA) was used for cDNA library construction according to the manufacturer's instructions. A Qubit 2.0 Fluorometer was used to determine the DNA content, and an Agilent 2100 bioanalyzer (CA, USA) was used to assess the integrity of the library DNA. Finally, quality-checked samples were selected for sequencing on the Illumina NovaSeq platform (Novogene, Beijing, China). After quality control, the raw sequencing data were subsequently analyzed using Hisat2 v2.0.5 and featureCounts v1.5.0, to conduct genome comparison and quantify gene expression levels. FPKM value of each gene was calculated. *P* < 0.05 and |log_2_ Fold Change|> 1 were used as the cutoffs for selecting the differentially expressed genes (DEGs) between the two groups.

### Cerebral ischemia disease (CID) network construction and analysis

Based on a previous study [30], a CID network was constructed by integrating the transcriptomic data with cerebrovascular disease-related genes. The effects of SalC on the CID disease network were further evaluated using the Efficiency of Recovery Regulation (EoR, Eq. (2)) method and Network Topology and Transcriptomics based Approach (NTRA) based on our previously published papers [30, 35, 36]. In particular, the EoR value was set to describe the recovery effect of the drug on each gene in this disease network, with 100% indicating complete recovery. The NTRA rank was used to determine the key genes involved in disease progression as well as drug effects, based on the transcriptomics and network topology information [30, 35, 36]. Pathway enrichment analysis was performed on the key genes in the CID network and on the DEGs in the transcriptomic data by Ingenuity Pathway Analysis (IPA).

2 $$EoR = 100\%-\left|100\%-\frac{Fold\,Change\,\left(\frac{SalC}{Model}\right)}{Fold\,Change \left(\frac{Sham}{Model}\right)}\right|.$$

### Real-time quantitative reverse transcription polymerase chain reaction assay (qRT-PCR)

The sequencing data were validated by qRT-PCR on a Bio-Rad CFX96 Touch™ Real-time PCR Detection System (Hercules, CA, USA). Equal amounts of RNA were extracted from each mouse in the same group and mixed at 2 μg. Mixed RNA samples were subjected to reverse transcription using the QuantiNova Reverse Transcription Kit (QIAGEN, Hilden, Germany). Gene expression was quantified using the SYBR Green PCR Kit (biosharp, Hefei, China) and specific primers (Sangon, Shanghai, China) on a CFX-Touch™ 96 Real-Time PCR System (Bio-Rad Laboratories, Hercules, CA, USA). The specific primer sequences are listed in Table 1. Taking *Actb* as the internal control, the relative gene expression was determined according to the 2^−ΔΔCt^ method. The experiment was repeated three times.

**Table 1:** Sequences of primers for q-PCR

Gene	Sense (5′–3′)	Anti-sense (5′–3′)
*Trem1*	ATGTGTTCACTCCTGTCATCAT	GAGAAGTCCACAGATGACTGAA
*Cxcl1*	ATGGCTGGGATTCACCTCAAGAAC	AGTGTGGCTATGACTTCGGTTTGG
*Il6*	CTCCCAACAGACCTGTCTATAC	CCATTGCACAACTCTTTTCTCA
*Actb*	CTACCTCATGAAGATCCTGACC	CACAGCTTCTCTTTGATGTCAC

Primers for mouse genes

### Western blot analysis

Total proteins were isolated from the ipsilateral ischemic hemisphere of mouse brain as well as from the BV2 cells, as previously described [37]. Protein concentrations were determined using a BCA Protein Assay kit (Thermo Fisher Waltham, MA, USA), and 15 or 20 μg of protein samples were separated by a 12% sodium dodecyl sulfate–polyacrylamide gel and electrophoretically transferred to polyvinylidene fluoride (PVDF) membranes (Merck Millipore, Darmstadt, Germany). After blocking with 5% non-fat milk, membranes were incubated with anti-TREM1 (1:500, 11791-1-AP), anti-TLR4 (1:500, 14358S), anti-p-NF-κB p65 (1:500, 3033 T), anti-NF-κB p65 (1:500, 8242 T), and anti-*β*-actin (1:1000, 4970S) primary antibodies overnight at 4 °C. After rinsing, membranes were incubated with horseradish peroxidase-conjugated secondary antibodies at room temperature for 1 h. After washing with Tris-buffered saline with Tween (TBST), the blots were visualized by an enhanced chemiluminescent substrate reagent (Bio-Rad, Hercules, CA, USA) and a ChemiDoc™ Imaging System (Bio-Rad, Hercules, CA, USA). Relative protein levels were analyzed using Image Lab software 6.0 and the experiment was repeated three times.

### Immunofluorescence staining

Paraffin-embedded brain sections were subjected to double immunofluorescence staining. Sections were heat repaired with a citrate buffer in a microwave and blocked with 5% bovine serum albumin (BSA) for 1 h. Subsequently, the primary antibody Iba-1 (1:100, ab178846, Abcam, Cambridge, MA, USA) was used for microglial detection [38] by an overnight incubation at 4 °C. The sections were then incubated with appropriate fluorescent secondary antibodies. The same protocol was used to visualize the expression of TREM-1(1:100, 11791-1-AP; Proteintech, Chicago, IL, USA). Finally, the sections were sealed with a fluorescent blocker containing 4′,6-Diamidin-2-phenylindol (DAPI, ZLI-9557, ORIGENE, China), and observed and photographed using an inverted fluorescence microscope (OLYMPUS, BX63, Tokyo, Japan) at 200 × magnification, with three samples per group. Three random microscopic fields in each section were analyzed.

### BV2 and HT22 cell cultures

Mouse microglial BV2 cells and hippocampal neuronal HT22 cells were purchased from the Cell Bank of the Type Culture Collection of the Chinese Academy of Sciences (Shanghai, China). Cells were cultured in Dulbecco’s Modified Eagle Medium (DMEM, Gibco, USA) supplemented with 10% heat-inactivated fetal bovine serum (FBS, Gibco, USA) and 1% penicillin–streptomycin mixture (100 × Penicillin–Streptomycin Solution, Gibco, USA) at 37 ℃ with 5% CO_2_ until 80% confluency before passaging.

### Oxygen-glucose-deprivation/reoxygenation (OGD/R) and drug treatment

To mimic cerebral ischemia/reperfusion (I/R) injury in vitro, HT22 or BV2 cells were subjected to OGD/R treatment. To initiate OGD/R, the culture medium was removed and the cells were washed three times with PBS. Cells were cultured in glucose- and FBS-free DMEM in an anaerobic chamber (Billups-Rothenberg, AR, USA) containing a gas mixture of 95% N_2_ and 5% O_2_ and transferred to an aerobic atmosphere (95% air and 5% CO_2_) for reoxygenation. The duration of hypoxia was 6 h for HT22 cells and 12 h for BV2 cells, respectively, followed by 2 h of reoxygenation. To determine the protective effect of SalC, cells were treated with 100 μM or 200 μM SalC during OGD/R treatment and underwent to cell viability assay.

### Establishment of a co-culture system of BV2 and HT22 cells by conditioned media

To determine the effect of SalC on microglia-neuron interactions during cerebral I/R injury, a co-culture system of BV2 and HT22 cells was established using conditioned medium. First, BV2 cells were exposed to OGD/R treatment as described above and then treated with or without two concentrations of SalC (100 and 200 μM) for 8 h. Second, the supernatant of BV2 cells from the OGD/R or SalC treatment was collected as conditioned medium for HT-22 cells. Third, after incubation with conditioned medium for 14 h, HT-22 cells were harvested for the cell viability assay.

### Determination of cell viability

The viability of HT-22 and BV2 cells was determined using a Cell Counting Kit-8 (CCK-8) assay. In brief, 10 μL CCK-8 reagent was added to the culture medium in each well and the cells were incubated for 2 h. The absorbance was measured at 450 nm using an Infinite M1000 Pro microplate reader (Tecan, Zurich, Switzerland).

### Statistical analysis

Data were analyzed by Graphpad prism 7 software and presented as mean ± standard error of the mean (SEM). Statistical differences were examined using one-way analysis of variance (ANOVA), followed by Dunnett's post hoc analysis or Student’s two-tailed t-test. *P* < 0.05 was considered statistically significant.

## Results

### SalC reduces cerebral infarction and improves neurological function in tMCAO mice

The infarct volume ratio in the mouse brain was evaluated to assess the neuroprotective effects of SalC in mice subjected to tMCAO. TTC staining showed that compared with the sham group, tMCAO caused a large infarct size in mouse brains, with a mean infarct volume ratio of 30.9% (Fig. 1C, D). Compared with the model group, both low- and high-dose SalC treatment (20 and 40 mg/kg, respectively) markedly reduced the volume of the cerebral infarct, exhibiting noticeable anti-ischemic activity (Fig. 1C, D). The average infarct volume ratios in the two groups were 15.5% and 10.9%, respectively. Notably, the effect of high-dose SalC (40 mg/kg) was comparable to that of edaravone, which is commonly used for the treatment of acute ischemic stroke [39]. Similar trends were observed in the neurological deficit scores, whereas high-dose SalC administration showed a comparable effect to edaravone in improving neurological function in tMCAO mice (Fig. 1E).

### SalC improves brain injury damage and neuronal apoptosis in tMCAO mice

**Fig. 2:**
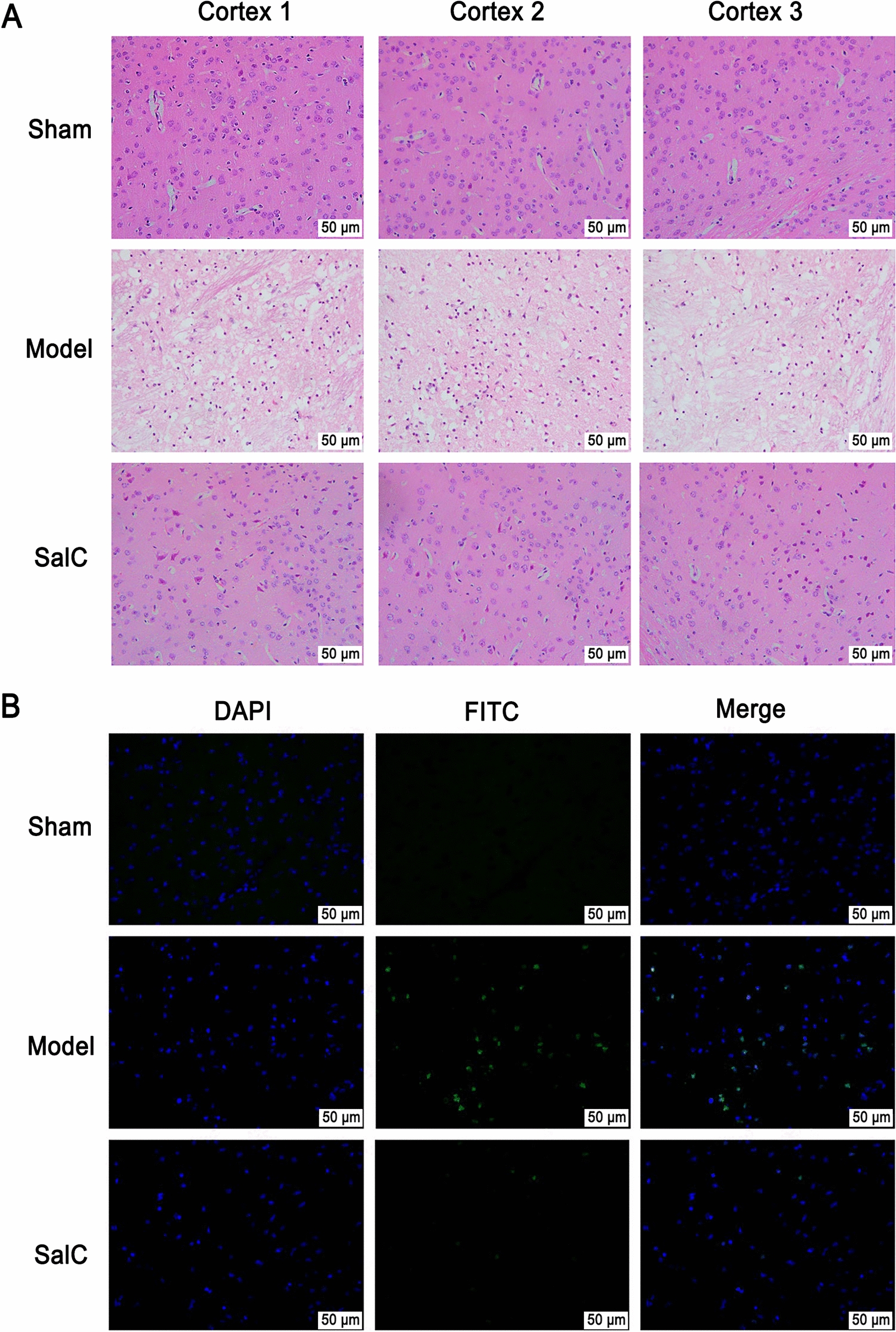
SalC reduces brain damage and neuron apoptosis in tMCAO mice. **A** H&E staining of three areas in the penumbra of the brain cortex. **B** TUNEL staining with representative photos showed apoptotic neurons in the penumbra of ischemic cortex (appearing yellow-green due to FITC labeling). n = 3, magnification of 400 ×

Histopathological examinations were performed on brain tissues using H&E staining. Noticeable histopathological changes were detected in the ipsilateral cortex of tMCAO mice compared to sham-operated mice, including numerous vacuoles, fragmented nuclei, and pyknosis (Fig. 2A). After high-dose SalC treatment, a marked reduction in histological damage was observed in the ischemic cortex. The effects of SalC on neuronal apoptosis were evaluated using the TUNEL assay (Fig. 2B). TUNEL-positive cells were noticeably increased after tMCAO than those in the sham group, while high-dose SalC treatment significantly reduced neuronal apoptosis in tMCAO mice. Taken together, these results suggest that SalC exerts a significant protective effect against acute cerebral ischemic injury in mice.

### SalC restores gene expression in the brain tissues of tMCAO mice

**Fig. 3:**
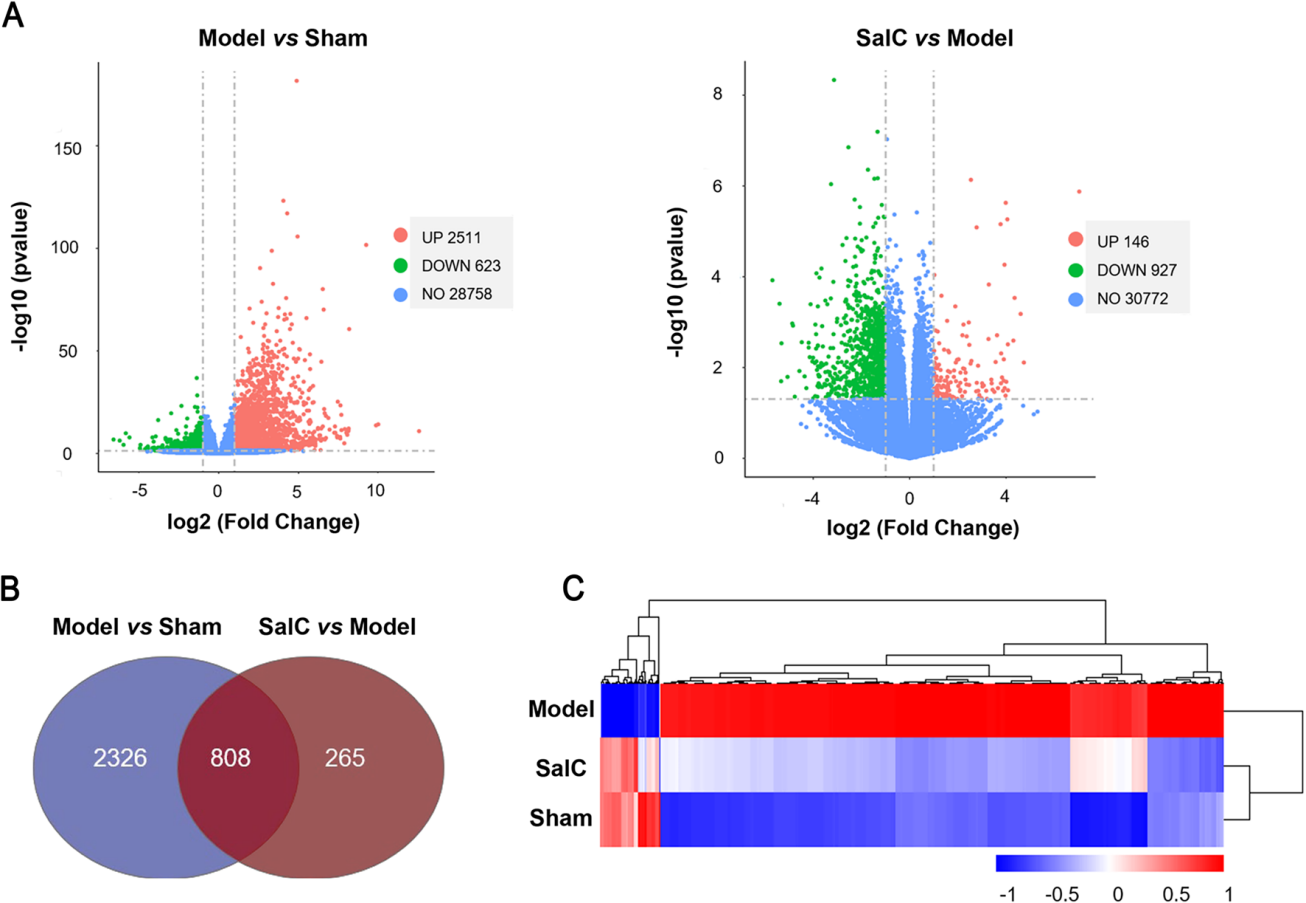
Transcriptional analysis shows that SalC restores gene expression in the brain tissues of tMCAO mice. **A** Volcano plot shows the DEGs. Genes with absolute fold changes > 2 and *P* value < 0.05 were highlighted in red and green, indicating up- and down-regulated genes, respectively. **B** Venn diagram of the DEGs. **C** Hierarchical clustering analysis of the 808 genes that were dysregulated under the modeling of tMCAO and reversed by SalC

To further investigate the mechanisms underlying the pharmacodynamic effects of SalC against acute ischemic stroke, transcriptome sequencing was performed on the ischemic penumbra of the mouse brain. DEGs were selected based on *P* < 0.05, |log_2_ Fold Change|> 1 between the model and sham groups, as well as between the SalC and model groups. Compared with the sham group, 3134 genes were differentially dysregulated in the model group, of which, 2511 genes were significantly upregulated and 623 genes were markedly downregulated (Fig. 3A). In contrast, 1073 genes were differentially expressed in the SalC group compared with the model group; of them, 927 genes were significantly downregulated and 146 genes were significantly upregulated. Moreover, 808 genes were common to the two DEG datasets (Fig. 3B). Hierarchical clustering analysis further showed that the 808 genes that were dysregulated in the tMCAO model were reversed by SalC treatment, which further demonstrated the protective effect of SalC against cerebral ischemic/reperfusion injury (Fig. 3C).

### SalC recovers the dysregulated CID network

**Fig. 4:**
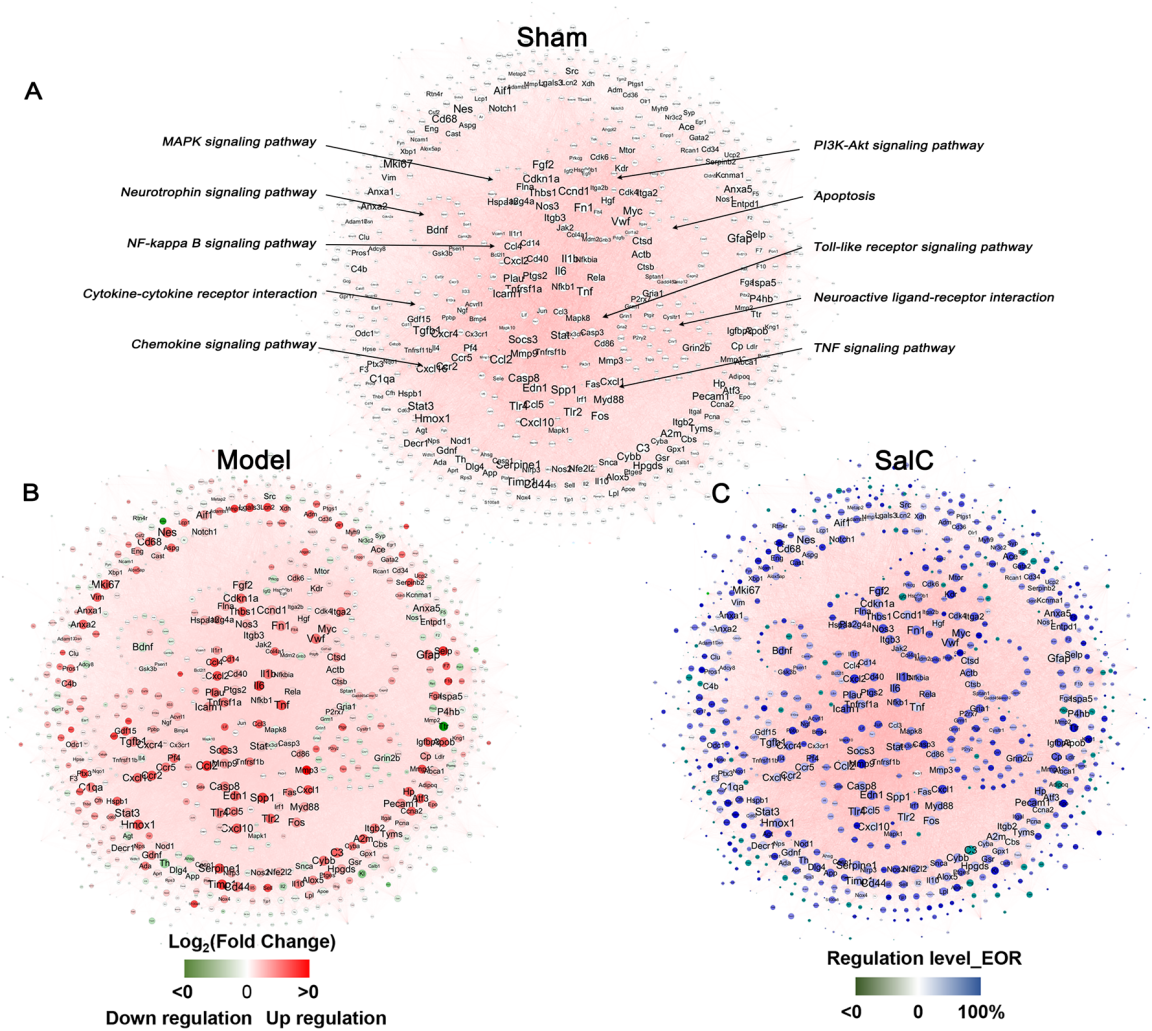
SalC recovers the unbalanced CID network disturbed by tMCAO modeling. **A** CID network. Node size represents NTRA rank. **B** Unbalanced CID network disturbed by tMCAO modeling. Red indicates log_2_ Fold Change (model/sham) > 0, representing upregulation of this gene expression in the model group compared with the sham group; Green indicates log_2_ Fold Change (model/sham) < 0, representing downregulation of this gene expression in the model group compared with the sham group. Moreover, the shade of the color indicates the degree of upregulation and downregulation. **C** Effect of SalC on the regulation of CID network. Blue represents the efficiency of EoR > 0, the darker the color, the greater the degree of regulation; Green represents EoR < 0, indicating that SalC has no regulatory effect on this gene. n = 4

According to our previously established method [40], a CID network was constructed using a combination of cerebrovascular disease-related genes and genes obtained from transcriptome sequencing, containing 864 nodes and 24,076 edges (Fig. 4A). In the network, node color (red: upregulation by tMCAO modeling; green: downregulation by tMCAO) was represented by log_2_ (fold change) to reflect gene expression changes between the model and sham groups (Fig. 4B), whereas the effect of SalC on disease-related genes was indicated by the EOR value (Fig. 4C). SalC recovered the dysregulated CID network caused by tMCAO modeling, with 80.0% (691/864) of the genes in the CID network displaying effective recovery regulation (Fig. 4C). Among them, 53.1% (367/691) of the genes exhibited more than 50% efficiency of recovery regulation (EoR > 50%), demonstrating the potent effect of SalC on cerebral ischemic injury. Additionally, these findings revealed that the protective effect of SalC is mediated through a multi-target mechanism.

### SalC exerts anti-cerebral ischemia effect by targeting neuroinflammation-related pathways

**Fig. 5:**
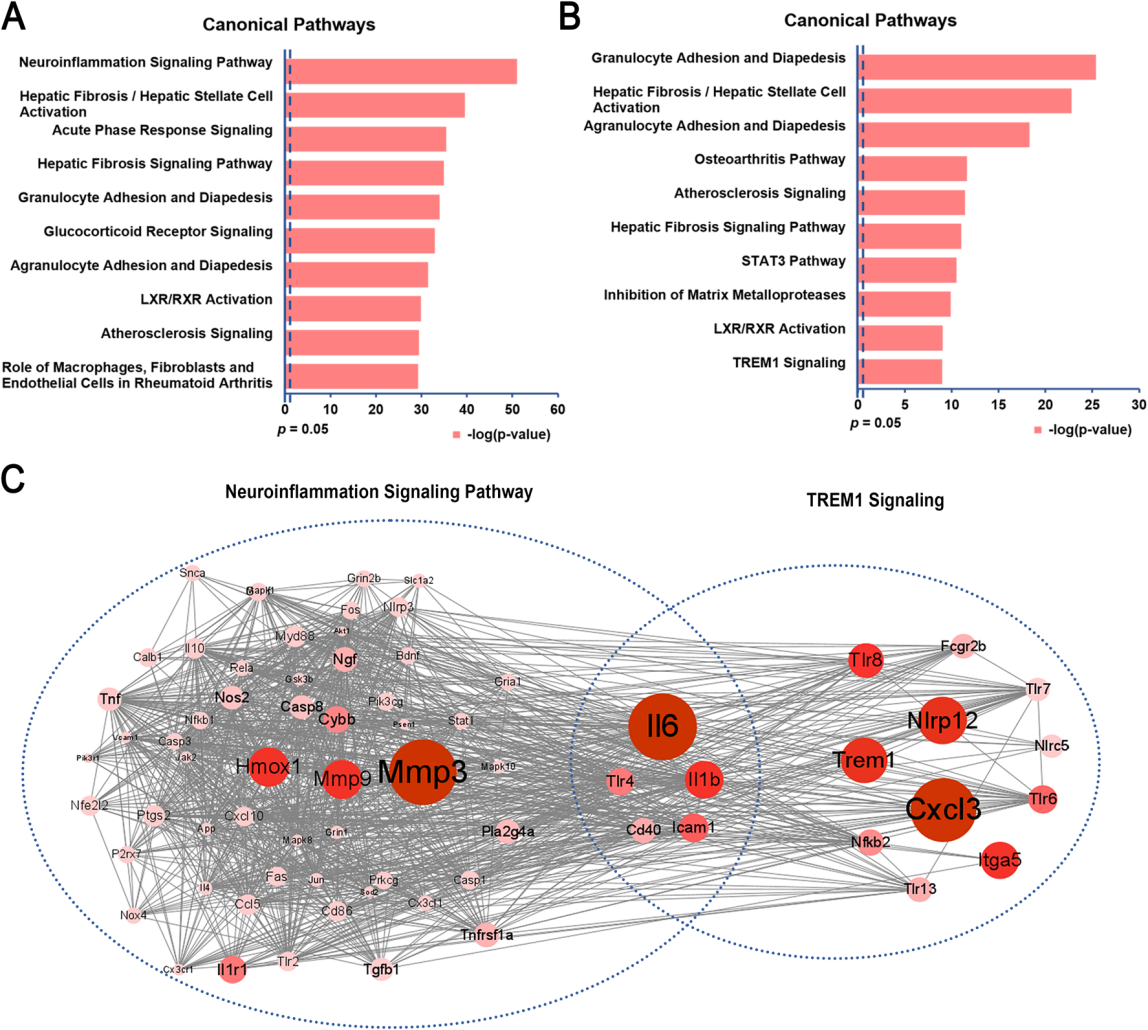
SalC mitigates stroke in mice by suppressing neuroinflammation and TREM1 signaling after tMCAO-induced cerebral ischemia. **A** The top 10 canonical pathways involved in the top 300 NTRA-ranked genes with EoR > 0. **B** The top 10 canonical pathways involved in the 808 DEGs. **C** Diagram of gene interactions in neuroinflammation and TREM1 signaling pathways. Node size and color shades represent the size of |log_2_ Fold Change|

To further explore the therapeutic mechanism of SalC, the NTRA rank was used to determine the key genes involved in disease progression and drug action based on transcriptomic and network topology information [30, 35, 36]. The top 300 NTRA-ranked genes were obtained, and pathway enrichment analysis was performed on 293 key genes with EoR > 0 among the top 300 genes. IPA revealed that the neuroinflammatory signaling pathway was significantly enriched in the top 1 pathway (*P* < 0.05, Fig. 5A); several inflammation-related pathways were also markedly affected, including acute phase response signaling, granulocyte adhesion, and agranulocyte adhesion and diapedesis (Fig. 5A). Pathway enrichment analysis was conducted on 808 common DEGs. Significant enrichment of the TREM1 signaling pathway was detected (Fig. 5B), which is closely related to inflammatory amplification [41]. Notably, several key genes involved in the TREM1 pathway were also affected in the neuroinflammation signaling pathway, acute phase response signaling pathway, granulocyte adhesion and diapedesis, and agranulocyte adhesion and diapedesis, including *Tlr4*, *Il6*, *Il1β*, and *Cxcl3*. By network approach, further joint analysis of TREM1 signaling pathway and neuroinflammation signaling pathway revealed that *Tlr4*, *Il6*, *Il1β*, *Cd40*, and *Icam1* were the common genes as well as the key nodes in these two pathways (Fig. 5C). TLR4 encoded by *Tlr4* gene is a member of the important Toll-like receptor family and functions to activate NF-κB signaling, which subsequently promotes the release of downstream inflammatory mediators and upregulates TREM1 expression [42]. The above evidence indicated that regulation of TLR4-TREM1-NF-κB signaling pathway might be an important mechanism in the action of SalC in acute ischemic stroke.

### Inhibition of neuroinflammation through the TLR4-TREM1-NF-κB signaling pathway is the main mechanism of SalC against ischemic stroke

**Fig. 6:**
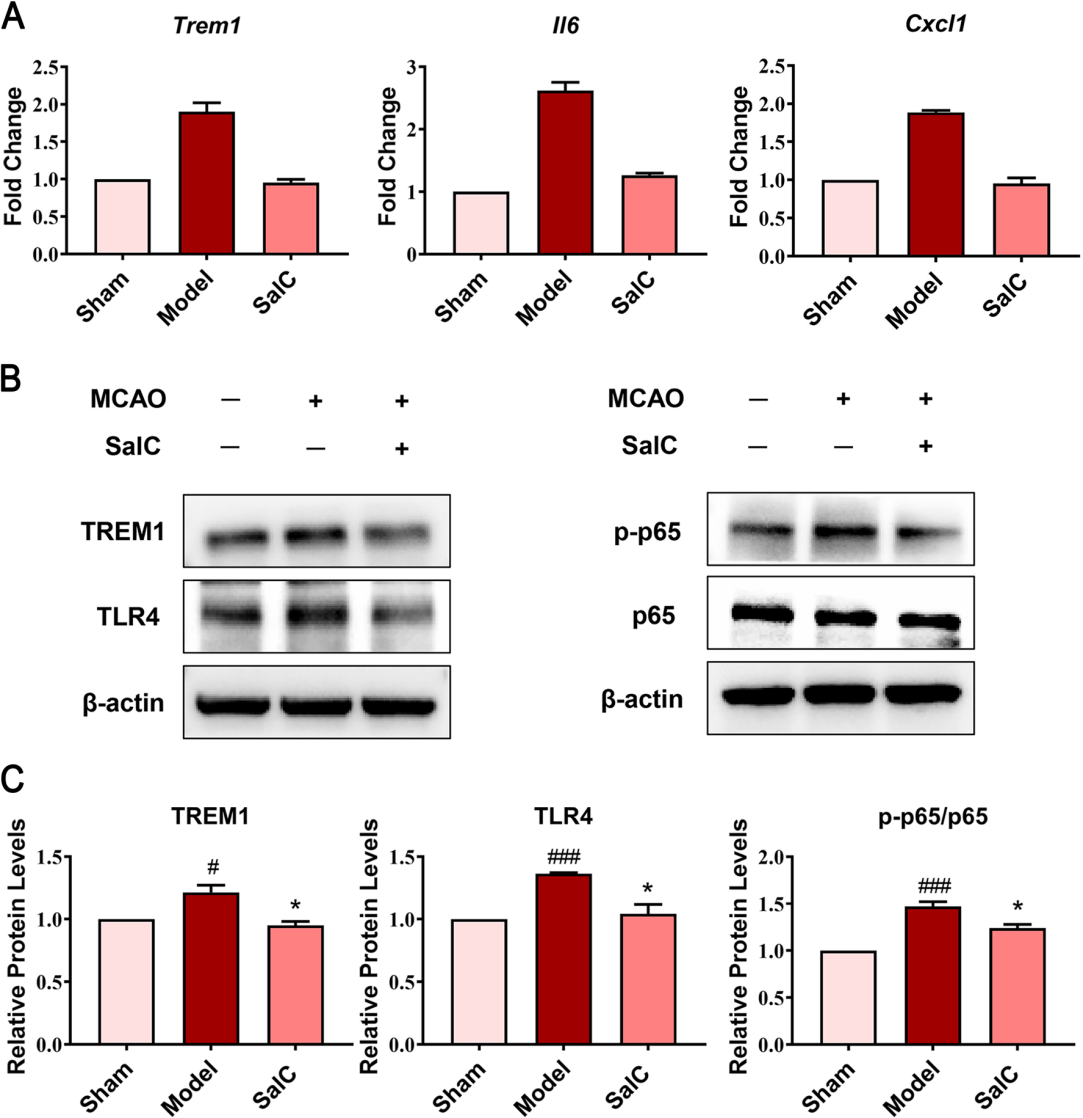
SalC attenuates cerebral ischemic stroke in tMCAO mice by inhibiting neuroinflammation via the TLR4-TREM1-NF-κB pathway. **A** The gene expressions of *Trem1*, *Il6*, and *Cxcl1* detected by qRT-PCR to validate transcriptome sequencing results. *β-actin* was used as an internal reference. **B** The protein expression levels of TREM1, TLR4, p-p65, and p65 in brain tissue were analyzed by western blot. β-actin was used as an internal standard. **C** Quantification was performed by Image Lab software. Data are expressed as mean ± SEM, and differences between groups were determined by t-test, n = 4. ^###^*P* < 0.001, ^#^*P* < 0.05 *versus* the sham group; *P < 0.05, versus the model group

The qRT-PCR and western blot were performed to further verify the regulation of TLR4-TREM1-NF-κB pathway by SalC. As shown in Fig. 6A, B, tMCAO modeling elevated the expression of *Trem1*, *IL6*, and *Cxcl1* in mouse brain tissue compared with sham-operated mice, while the expression of these genes was effectively downregulated by SalC administration. Consistently, the expression of TREM1, TLR4, and phosphorylated p65 was significantly upregulated in the brain tissue of tMCAO mice compared with that in sham surgery mice, which was inhibited by SalC treatment (Fig. 6C, D). These results suggest that SalC could attenuate ischemic stroke through the TLR4-TREM1-NF-κB signaling pathway.

### SalC inhibited the expression of TREM1 in brain microglia of tMCAO mice

**Fig. 7:**
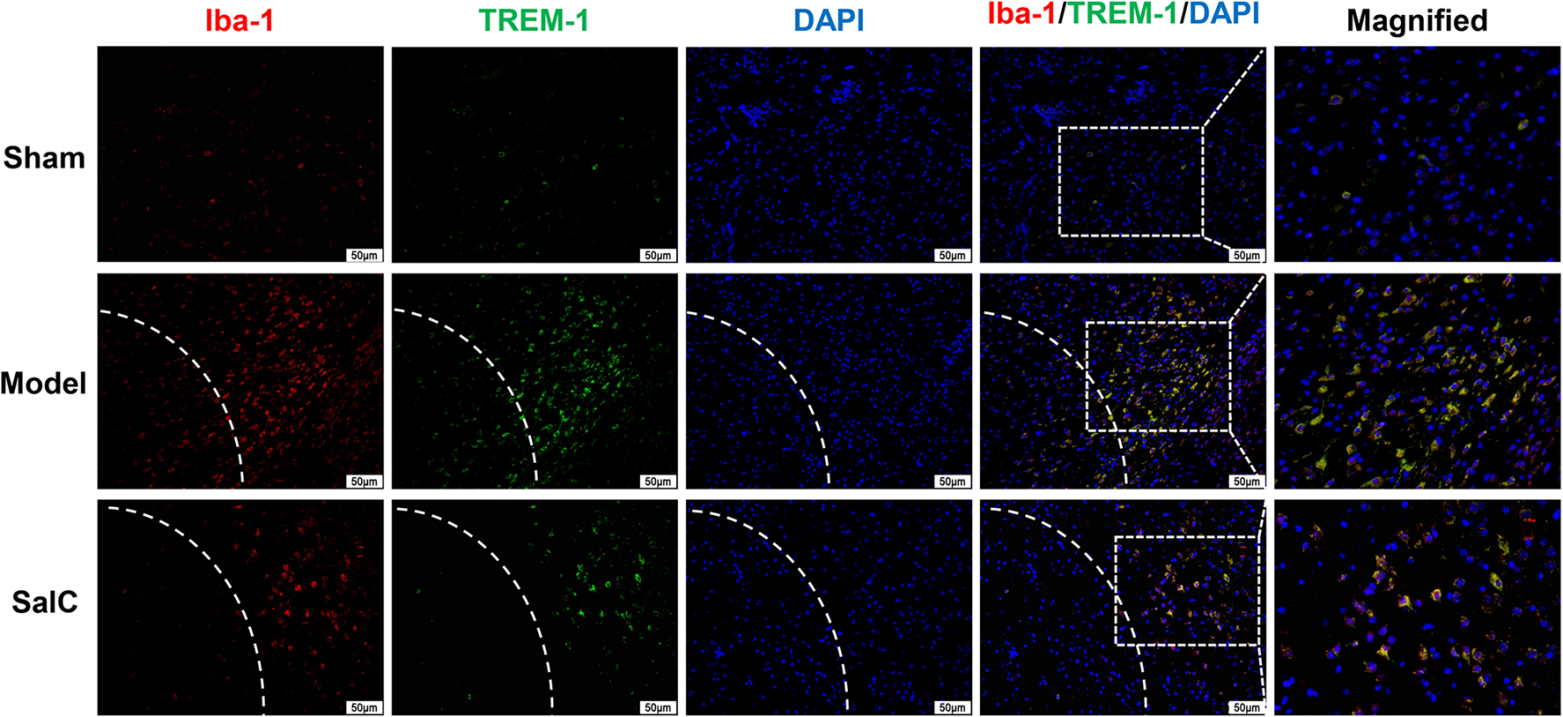
SalC inhibits TREM1 expression in microglia. Representative double-staining immunofluorescence of Iba-1 (red) and TREM1 (green) in the ischemic transition region of brain sections. Nuclei were stained with DAPI (blue). n = 3, magnification 200 ×

As the overexpression of TREM1 in microglia contributes to post-stroke neuroinflammatory damage [18], its expression in the microglia of tMCAO mice after SalC administration was investigated. Iba-1, a microglia/macrophage-specific marker, was used for microglial detection. Immunofluorescence staining showed that the number of TREM1-positive microglia was noticeably increased in the penumbra region after tMCAO compared with that in the sham group (Fig. 7), indicating that ischemia/reperfusion brain injury triggered the upregulation of TREM1 expression in microglia. In contrast, the expression of TREM1 in microglia was significantly decreased after SalC administration compared with that in the model group, suggesting that SalC inhibits the overexpression of TREM1 in microglia within the ischemic penumbra, and thus exerts its anti-neuroinflammatory effect on ischemic stroke.

### Inhibition of TLR4-TREM1-NF-κB pathway is an important mechanism of SalC against OGD/R injury

**Fig. 8:**
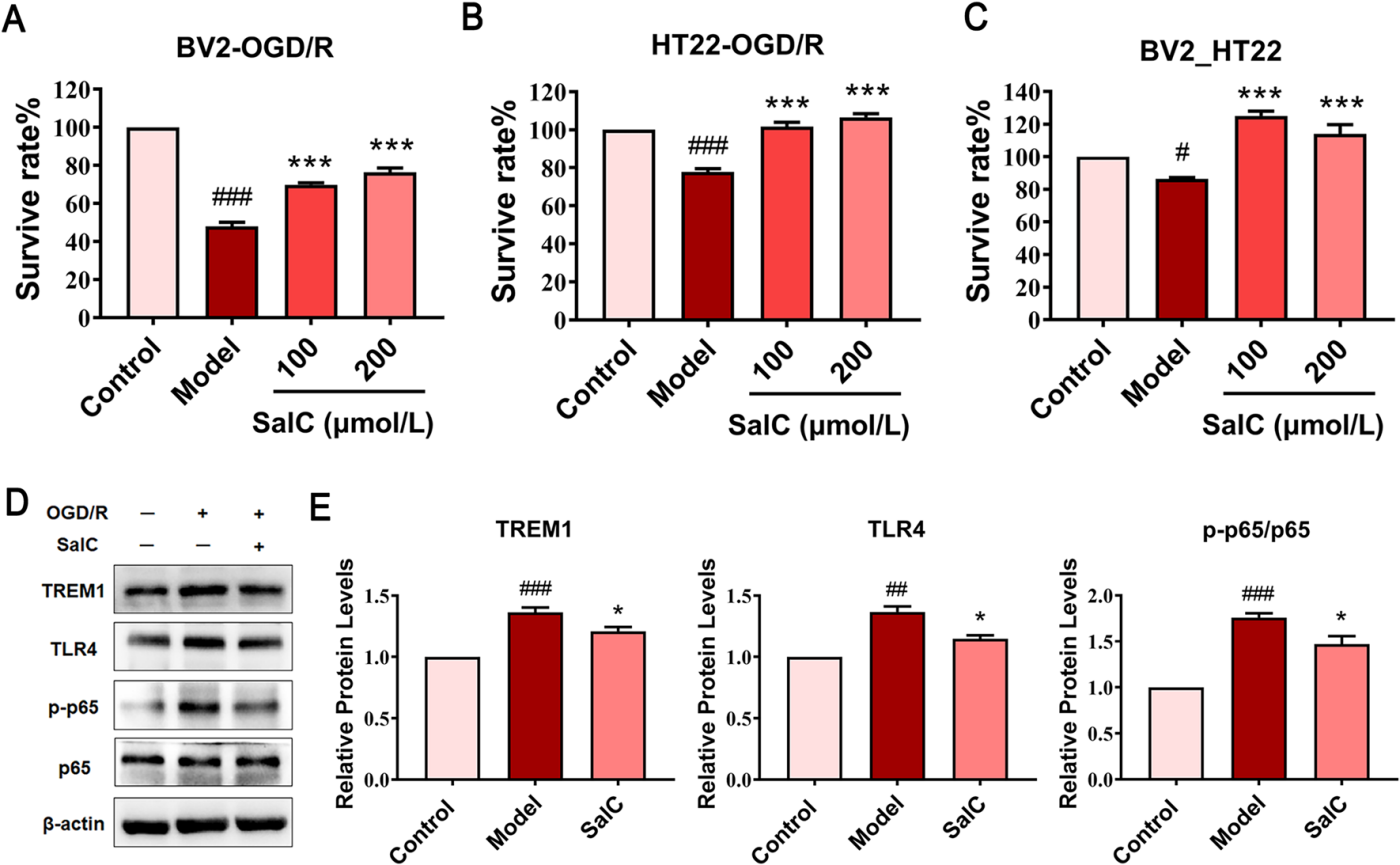
The protective effect of SalC on OGD/R injury in vitro. **A** Effects of SalC on OGD/R modeling of BV2 cells. **B** Effects of SalC on OGD/R modeling of HT22 cells. **C** Effects of SalC on the co-culture system of BV2 and HT22 cells. **D** The protein expression levels of TREM1, TLR4, p-p65 and p65 in BV2 cells were analyzed by western blot. β-actin was used as an internal standard. **E** Quantification was performed by Image Lab software. Data are expressed as mean ± SEM, and differences between groups were determined by t-test, n = 3. ^###^*P* < 0.001, ^##^*P* < 0.01, ^#^*P* < 0.05, *versus* the sham group; ***P < 0.001, **P < 0.01, *P < 0.05, versus the model group

The effects of SalC were evaluated using a co-culture system of BV2 and HT22 cells in conditioned media under OGD/R conditions. The survival rate of OGD/R-injured BV2 and HT22 cells was significantly increased by SalC treatment in a dose-dependent manner compared with that of the model group (Fig. 8A, B). To further determine the role of microglia-mediated inflammation in neurons under hypoxic conditions, the supernatant of BV2 cells (OGD/R + SalC) was collected as a conditioned medium to co-culture with HT22 cells. We found that the supernatant of post-modeling BV-2 cells caused a decrease in the survival rate of HT22 cells compared with the supernatant of normal cultured microglia, whereas SalC treatment significantly attenuated this trend (Fig. 8C). These results suggest that the detrimental effects of hypoxic microglia on neuronal survival through the secretion of soluble substances can be alleviated by SalC.

Finally, western blotting was performed to verify the inhibitory effect of SalC on the TREM1 signaling pathway in microglia. The expression of TREM1, TLR4, and p-p65/p65 in microglia significantly increased after exposure to OGD/R, whereas the expression of these proteins was significantly downregulated by SalC administration (Fig. 8D, E). These results were consistent with in vivo studies, demonstrating that SalC indeed exerted protective effect on cerebral ischemic/reperfusion injury by inhibiting the activation of TLR4-TREM1-NF-κB signaling pathway in microglia.

## Discussion

Acute stroke is a difficult-to-treat disease with high morbidity, disability, and mortality rates that seriously endangers human health [6, 43]. Tissue fibrinogen activator (tPA) is currently the only effective drug officially approved by the FDA for the treatment of acute ischemic stroke; however, its clinical application is greatly limited because of its narrow therapeutic window [44]. Recently, drugs for the treatment of acute ischemic stroke have become the focus of drug development. Acute ischemic stroke is associated with diverse factors such as microglia-neuron interactions, inflammation, oxidative stress, excitotoxicity, apoptosis, and autophagy [45, 46]. There is growing evidence that modulating the expression of crucial molecules in the early stages of stroke can serve as an effective strategy for preventing neuronal damage [47, 48]. These findings highlight the significance of this study.

A major finding of this study is that SalC protects against the early stages of stroke. Although modern pharmacological studies have found that SalC possesses pharmacological activities such as antioxidant, neuroinflammation inhibition, and anti-apoptotic activities, further suggesting that it might have potential as a treatment for ischemic cardiovascular and cerebrovascular diseases, the relationship among SalC, microglia, and stroke remains unclear. Our findings showed that SalC treatment significantly counteracted tMCAO injury in vivo and protected cells against OGD/R-induced cell death in vitro. Moreover, based on integrative network pharmacology and whole transcriptome sequencing, our results suggest that SalC exhibits global modulation of multitarget activity in the CID network during the early stages of stroke. The most significant of them, the protective effects of SalC, were achieved via the inhibition of the TLR4-TREM1-NF-κB pathway, which further inhibited neuroinflammation.

Microglia play an indispensable role in neuroinflammation. Inflammatory mediators produced by microglia, such as pro-inflammatory cytokines (TNF-α, IL-1β, and IL-6), significantly promote neuroinflammation, thereby mediating stroke. Notably, TREM1 plays a vital role in the neuroinflammatory response of microglia [49]. Activation of TREM1 triggers microglia to secrete pro-inflammatory chemokines and cytokines such as IL-6 and IL-1β, thereby amplifying related inflammation [18, 50]. TREM1 expression is elevated during acute ischemic stroke, and blocking TREM1 expression can reduce the extent of infarction and neuronal damage [18]. This study found that both gene and protein expression of TREM1 and pro-inflammatory cytokines were upregulated in the tMCAO mouse model and microglia, but were significantly downregulated by SalC treatment (Figs. 6, 7, 8). This suggests that SalC may inhibit pro-inflammatory factors in microglia through the TREM1 pathway, thereby suppressing neuroinflammation and exerting a protective effect against stroke. Concurrently, in stroke, the peripheral immune system responds by activating immune cells that interact with the central immune system. The systemic effects of stroke, including the activation of peripheral immune cells, significantly affect its progression and severity. TREM1 surface expression has been reported to be induced in monocyte/macropahge subsets in the spleen and blood within hours of cerebral ischemia; together with neutrophils expressing high levels of TREM1, these myeloid cells accumulated in ischemic brain and increased cerebral injury and motor deficits [51]. Thus, by regulating TREM1 activity in both the peripheral and central systems, it may be possible to diminish the inflammatory response, thereby reducing the damage caused by stroke [52, 53]. It has been demonstrated that SalC can attenuate NF-κB mediated inflammatory responses in both whole animals and microglia [54]. Therefore, we postulated that SalC may have the potential to regulate peripheral and microglial TREM1 expression, thereby alleviating neuroinflammatory responses.

Additionally, studies have found that TLR signaling pathways are closely related to cerebral ischemic diseases [12, 55]. As transmembrane pattern recognition receptors, TLRs have various subtypes, from TLR1 to TLR11, which are capable of identifying highly conserved structures in pathogens and molecular patterns associated with endogenous damage [55]. TLR4 is closely associated with acute ischemic stroke severity. Early blocking of TLR4 in rodent models and in vitro human ischemic brain models can reduce the inflammatory response and oxidative stress caused by ischemic brain injury [56]. In the context of ischemic stroke, the activation of TLR4 leads to the translocation of NF-κB to the nucleus, promoting the transcription of genes related to inflammation and immune responses. Studies have highlighted the potential benefits of early blockade of TLR4, which can reduce oxidative stress and alleviate inflammation mediated by the NF-κB pathway [56]. By inhibiting TLR4, it may be possible to reduce the detrimental effects of inflammation mediated by NF-κB. Consistently, our experimental results showed significant upregulation of TLR4 and phosphorylated p65 expression in the brain tissue of mice in the tMCAO model group, which was significantly inhibited by SalC administration.

TREM1 and TLR4 synergistically interact to enhance inflammatory responses, and co-activation is crucial for understanding the pathophysiology of stroke and potential therapeutic interventions. Studies have shown that TREM1 amplifies inflammatory responses mainly through synergistic pattern recognition receptors, such as TLRs and Nod-like receptors [42]. TLR4 activates NF-κB signaling, leading to the release of downstream inflammatory mediators and upregulation of TREM1 expression. Notably, the released TREM1 can bind to DAP12, thereby activating NF-κB and promoting the expression of downstreaming pro-inflammatory cytokines, chemokines as well as cell surface receptor molecules, thereby enhancing the inflammatory response [42]. Studies have shown that TREM1 interacts with the TLR4 receptor complex or is a component of this complex, and that specific inhibitors of TLR4 can downregulate the cross-linking effect of TREM1 [57]. Similar studies investigated the functional genomics of silenced TREM-1 and its impact on TLR4 signaling in macrophages. These findings indicate that silencing TREM-1 regulates the expression of certain cytokines and receptors, affecting the TLR4-mediated signaling pathway. This suggests that TREM1 can amplify TLR4 signaling, influencing the overall inflammatory response [58]. Together, these studies highlight the close connection between TREM1 and TLR4 in inflammatory responses and the importance of the TLR4-TREM1-NF-κB pathway in stroke disease. Our study demonstrated the pro-inflammatory role of the TLR4-TREM1-NF-κB pathway in early acute stroke. Furthermore, a significant trend toward the recovery of these phenotypes and related genes after SalC treatment was observed. This suggests that SalC can inhibit stroke-related neuroinflammation, and thus plays a role in early intervention in ischemic stroke.

Notably, our results confirmed the impact of SalC on microglia-neuron interactions during brain I/R injury (Fig. 8), consistent with reports of dysregulated protein expression levels of TREM1, TLR4, and p-p65/p65 in the tMCAO model. These results provide evidence that SalC mitigates ischemic stroke in vivo by inhibiting the TLR4-TREM1-NF-κB pathway and blocking neuroinflammation, suggesting that SalC is a promising new drug for the treatment of early ischemic stroke.

## Conclusions

**Fig. 9:**
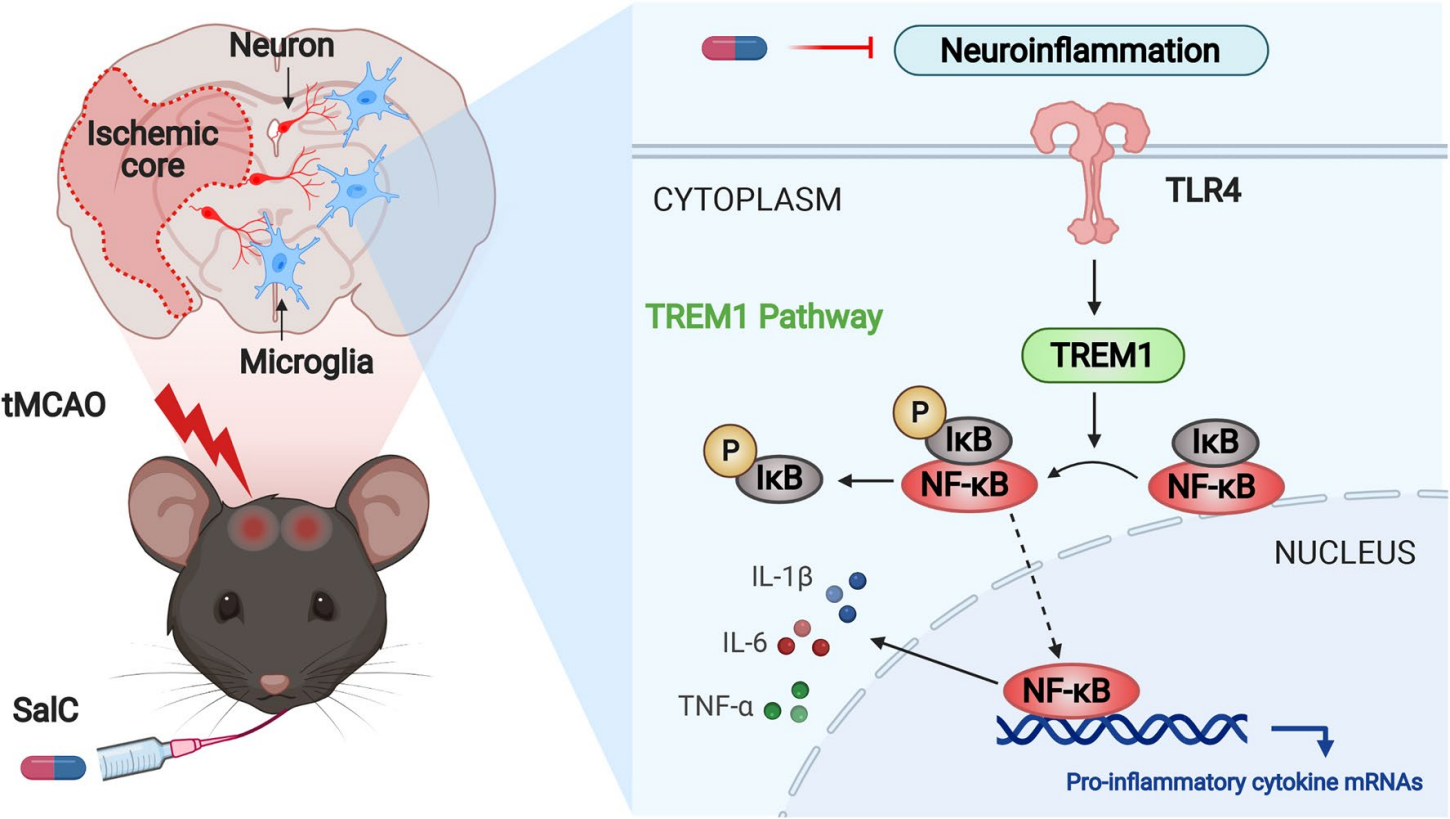
The scheme of the mechanism for the protective effects of SalC on cerebral ischemia stroke in tMCAO mice by inhibiting neuroinflammation via the TLR4-TREM1-NF-κB pathway

In conclusion, this study provides evidence that SalC exerts a notable protective effect against early cerebral ischemic injury by mitigating neuroinflammation. The underlying mechanism involves the down-regulation of TREM1 expression on microglia, leading to the inhibition of the TLR4-TREM1-NF-κB pathway. These findings suggest that SalC is a promising potential therapeutic agent for early-stage ischemic stroke. (Fig. 9). Further research is required to explore the interaction between the peripheral and brain immune systems, which contributes to neuroinflammation and activates the TLR4-TREM1-NF-κB pathway. Moreover, owing to the heterogeneity of microglia, our understanding of their function in ischemic stroke is limited. Therefore, employing single-cell sequencing technology is necessary for a more detailed elucidation of the roles and effects of SalC.

## Data Availability

The data that support the findings of this study are available from the corresponding author upon reasonable request.

## Abbreviations

ANOVA: Analysis of variance
BSA: Bovine serum albumin
CCK-8: Cell Counting Kit-8
CNS: Central nervous system
CID: Cerebral ischemia disease
CCA: Common carotid artery
DEGs: Differentially expressed genes
DMEM: Dulbecco’s Modified Eagle Medium
EoR: Efficiency of Recovery Regulation
ECA: External carotid artery
FBS: Fetal bovine serum
FDA: Food and Drug Administration
HE: Hematoxylin–eosin
ICA: Internal carotid artery
IPP: Image-Pro Plus
I/R: Ischemia/reperfusion
MCA: Middle cerebral artery
OGD/R: Oxygen-glucose-deprivation/reoxygenation
PVDF: Polyvinylidene fluoride
qRT-PCR: Real-time quantitative reverse transcription polymerase chain reaction assay
r-tPA: Recombinant tissue plasminogen activator
SalC: Salvianolic acid C
SEM: Standard error of the mean
TUNEL: Terminal deoxynucleotidyl transferase mediated dNTP nick end labeling
tMCAO: Transient middle cerebral artery occlusion
TREM1: Triggering receptor expressed on myeloid cells 1
TBST: Tris-buffered saline with Tween
TTC: 2,3,5-Triphenyltetrazolium chloride
